# BAN Score and Distinct Early Cardiometabolic Risk Signatures in a Non-Diabetic Population: A Cross-Sectional Analysis

**DOI:** 10.3390/healthcare13182384

**Published:** 2025-09-22

**Authors:** Yazeed Alshuweishi, Noha A. Alshuwayer, Lama Izziddeen, Arwa Abudawood, Dalal Alfayez, Ahmed M. Basudan

**Affiliations:** 1 Chair of Medical and Molecular Genetics Research, Department of Clinical Laboratory Sciences, College of Applied Medical Sciences, King Saud University, Riyadh 12372, Saudi Arabia; 2 Department of Anatomy, College of Medicine, King Saud University, P.O. Box 2925, Riyadh 11461, Saudi Arabia; nohamd@ksu.edu.sa; 3 Department of Clinical Laboratory Sciences, College of Applied Medical Sciences, King Saud University, Riyadh 12372, Saudi Arabia; 4 Department of Family and Community Medicine, Prince Sultan Military Medical City, Riyadh 11159, Saudi Arabia; aabudawood@psmmc.med.sa (A.A.);

**Keywords:** BAN score, blood pressure, cardiovascular risk, dysglycemia

## Abstract

**Background:** The BMI–albumin–neutrophil-to-lymphocyte (BAN) score integrates adiposity, nutritional status, and systemic inflammation, but its role in detecting early cardiometabolic changes remains unclear. This study examined associations of the BAN score with vascular, glycemic, and lipid markers in non-diabetic adults. **Methods:** This retrospective cross-sectional study included 162 non-diabetic subjects. Associations between the BAN score and vascular, glycemic, and lipid parameters were examined using Spearman’s correlation, ROC analysis, and regression models adjusted for age, sex, smoking status, and medication use. **Results:** Patients had a median age of 37 years, 72.8% were female, with median BMI 33 kg/m^2^, albumin 4.4 g/dL, and NLR 1.3. Higher BAN scores correlated with systolic blood pressure (SBP) (r = 0.23, *p* < 0.01), pulse pressure (PP) (r = 0.26, *p* < 0.001), and HbA1c (r = 0.22, *p* < 0.01). Compared with the lowest tertile, higher BAN tertiles showed significantly elevated SBP, PP, and HbA1c (*p* < 0.01). In adjusted models, each one-unit increase in BAN score was associated with higher SBP (β = 1.01, *p* = 0.037), PP (β = 0.66, *p* = 0.006), and HbA1c (β = 1.85, *p* = 0.008). No associations were found with the atherogenic index of plasma (AIP), Castelli risk index I (CRI-I), or Castelli risk index II (CRI-II). ROC analysis showed moderate discriminative ability for hypertension (AUC = 0.66) and HbA1c (AUC = 0.65). **Conclusions:** The BAN score is associated with a distinctive early cardiometabolic risk, particularly elevated SBP, widened PP, and early glycemic alterations. Further research should define the BAN score’s mechanisms and preventive utility.

## 1. Introduction

Cardiovascular diseases (CVDs) comprise a broad spectrum of pathologies involving the heart and vasculature and persist as the predominant global cause of premature mortality and long-term morbidity [1]. This group includes hypertensive heart disease, coronary artery disease, cerebrovascular conditions such as stroke, heart failure, and other forms of cardiac dysfunction [2,3]. According to the most recent Global Burden of Disease projections, cardiovascular deaths are expected to rise from 20.5 million in 2025 to 35.6 million by 2050, with ischemic heart disease remaining the leading cause and high systolic blood pressure the primary risk factor [4]. The mortality rate from CVDs in Saudi Arabia was 203.6 deaths per 100,000 people in 2021, with ischemic heart disease as the leading cause [5]. This escalating public health issue underscores the urgent need for comprehensive, evidence-based strategies to prevent, detect early, and manage CVDs at both national and international levels [6].

The substantial burden of CVDs largely stems from interconnected metabolic and clinical conditions that significantly raise the risk of illness and death. A critical contributor is metabolic syndrome (MetS), a multifactorial disorder marked by the coexistence of insulin resistance, central adiposity, elevated blood pressure, abnormal lipid profiles, and reduced glucose tolerance [7,8,9,10]. Global health authorities such as the World Health Organization (WHO) as well as the National Cholesterol Education Program (NCEP) have outlined diagnostic criteria for MetS, including additional indicators like microalbuminuria and elevated plasma glucose or insulin levels to assess cardiovascular risk [7,9]. Insulin resistance, a central feature of MetS, contributes to vascular dysfunction via mechanisms such as reduced vasodilation, enhanced sodium reabsorption, and stimulation of the renin–angiotensin–aldosterone system (RAAS). Inflammation from adipose tissue exacerbates this effect by releasing pro-inflammatory cytokines and free fatty acids, thereby disrupting normal glucose and lipid metabolism. Hyperinsulinemia also triggers the sympathetic nervous system, which further promotes hypertension [11,12,13,14]. Moreover, dyslipidemia, characterized by elevated concentrations of low-density lipoprotein (LDL) and triglycerides alongside decreased high-density lipoprotein (HDL) levels, further contributes to cardiac injury. Lipid accumulation within cardiomyocytes leads to oxidative stress, mitochondrial dysfunction, and reduced contractile efficiency, while circulating lipid imbalances further accelerate the development of coronary heart disease [12]. Among these interrelated risk factors, hypertension stands out as a particularly insidious threat, often asymptomatic in its early stage. It functions as a silent form of CVD, causing progressive vascular and organ damage without warning signs. Its global prevalence has more than twofold in the last thirty years, increasing from approximately 650 million cases in 1990 to over 1.3 billion by 2019 [14]. As a hallmark of cardiometabolic dysfunction, hypertension amplifies the adverse impact of obesity, inflammation, and dyslipidemia [15]. Early identification of individuals at high risk enables prompt intervention, thereby minimizing long-term complications and lowering mortality rates [16].

Given these overlapping mechanisms of obesity, inflammation, and dyslipidemia, there is growing interest in composite biomarkers that capture multiple dimensions of cardiometabolic risk. One such index is the BAN score, which integrates body mass index (BMI), serum albumin, and neutrophil-to-lymphocyte ratio (NLR). This score has emerged as a promising marker that integrates anthropometric, nutritional, and inflammatory dimensions into a single index. By combining body mass index (BMI), serum albumin, and neutrophil-to-lymphocyte ratio (NLR), the BAN score captures three interrelated domains of health: obesity and adiposity burden, nutritional and hepatic synthetic status, and systemic inflammatory response. This multidimensionality represents a key strength, as cardiometabolic dysfunction rarely stems from a single isolated factor. Originally developed in oncology research, the BAN score was designed to improve risk stratification by addressing the recognized impact of malnutrition and inflammation on long-term outcomes [17,18]. Its predictive utility has since been validated in several cancer cohorts, where higher BAN scores consistently correlated with poorer survival and greater disease progression [17,18]. Despite this evidence, applications of the BAN score remain largely restricted to oncology settings. This represents a missed opportunity, given that chronic low-grade inflammation, metabolic dysregulation, and nutritional imbalances are hallmarks of cardiometabolic diseases as well. Importantly, all three components of the BAN score have independent associations with cardiovascular outcomes: elevated BMI is a well-established risk factor for hypertension and dyslipidemia [19], hypoalbuminemia reflects catabolic and pro-inflammatory states that worsen vascular health [20,21], and higher NLR indicates systemic inflammation linked to atherosclerosis and insulin resistance [22,23]. By consolidating these parameters, the BAN score may provide a more holistic and clinically practical tool for identifying individuals at heightened cardiometabolic risk.

Extending the use of the BAN score into cardiovascular and metabolic contexts, particularly among non-diabetic adults, addresses an important gap in the literature. This study seeks to examine the relationship between the BAN score and early indicators of cardiometabolic risk among adults without diabetes. Specifically, the analysis focuses on how the BAN score correlates with blood pressure components, HbA1c levels, and lipid ratios, intending to determine its potential utility as an early indicator of cardiometabolic stress in individuals without diagnosed diabetes.

## 2. Methods

### 2.1. Study Design

This is a retrospective investigation was carried out at Prince Sultan Military Medical City (PSMMC) in Riyadh, Saudi Arabia. Ethical clearance was granted by the Institutional Review Board of the Scientific Research Center at PSMMC (IRB Approval No: E-2568; approval date 11 May 2025). Data were extracted between May and June 2025 from digital health records of adult patients who attended outpatient clinics from January to April 2025, identifying 162 non-diabetic subjects through the Family Medicine Lifestyle Clinic. For each patient, demographic information, anthropometric measures, and laboratory biomarkers were obtained from a single clinical encounter to ensure consistency. Biomarkers, including serum albumin, complete blood count–derived indices, and metabolic parameters, were analyzed from blood samples collected during that same visit, thereby minimizing variability related to timing of measurements. Eligible subjects for inclusion were adults aged 18 years or older with complete laboratory profiles. Those excluded comprised individuals under 18 years of age, pregnant women, and patients lacking full or adequate laboratory data.

Prehypertension, also referred to as elevated blood pressure, was classified as Systolic blood pressure (SBP) of 120–129 mmHg with a diastolic blood pressure (DBP) ≤ 80 mmHg in individuals without a documented hypertension diagnosis or antihypertensive therapy [24]. The definition of hypertension included any of the following: (1) SBP ≥ 130 mmHg and/or DBP ≥ 90 mmHg measured at the clinic; (2) prior documented diagnosis of hypertension; or (3) active treatment with antihypertensive drugs [24]. Pulse pressure (PP) was determined by subtracting DBP from SBP, with values of 40 mmHg or higher classified as elevated [25]. Mean arterial pressure (MAP) was derived using the formula as MAP = DBP + ⅓(SBP − DBP), with values ≥100 mmHg indicating increased cardiovascular risk [26]. The atherogenic index of plasma (AIP) was computed as log_10_(TG/HDL-C), with values >0.24 considered atherogenic [27]. Castelli risk indices were computed, with CRI-I defined as TC/HDL-C and CRI-II defined as LDL-C/HDL-C, using cut-off values of >5 and >3, respectively [27]. Glycated hemoglobin (HbA1c) was assessed, with values ≥5.7% indicating early glycemic abnormalities consistent with prediabetes [28].

### 2.2. Data Analysis

Normality of continuous variables was evaluated using the Shapiro–Wilk test. Because most were non-normal, data are presented as median (±IQR) and compared across BAN score tertiles using the Kruskal–Wallis test with Dunn’s post hoc adjustment. Spearman’s rank correlation assessed associations between BAN score and hemodynamic (SBP, DBP, PP, MAP), glycemic (HbA1c), and lipid indices (AIP, CRI-I, CRI-II). Discrimination for predefined abnormal states was evaluated using ROC analysis. Linear regression was applied to estimate the effect of each one-unit increase in BAN score, with Model 1 unadjusted and Model 2 adjusted for age and sex, presenting β coefficients alongside 95% confidence intervals. Risk evaluation was performed through calculations of prevalence risk (PR) and odds ratios (OR). All analyses were conducted using GraphPad Prism version 9.0 (GraphPad Software, San Diego, CA, USA), with statistical significance defined as *p* < 0.05.

## 3. Results

### 3.1. Clinical Features and Comorbid Status

As shown in Table 1, a total of 162 non-diabetic participants were included, with a median age of 37 (±28–45) years and predominantly female (72.8%). The median BMI was 33 (±27–36) kg/m^2^, serum albumin 4.4 (±4.2–4.6) g/dL, NLR 1.3 (±0.87–1.92), and WBC count 6.4 (±5.3–7.8) × 10^9^/L. Comorbidities included hypothyroidism (13.0%) and PCOS (5.6%), while 6.8% were on antihypertensive therapy, 9.9% were smokers, 21.6% reported iron supplementation, and 17.3% were receiving GLP-1 receptor agonists.

### 3.2. Comparison of Clinical and Biochemical Parameters Across BAN Score Tertiles

Subjects were stratified into tertiles based on the BAN score, revealing significant differences across several clinical and laboratory parameters (Table 2). SBP was higher in the second tertile and third tertiles (124 and 120 mmHg, respectively) compared to the first tertile (117 mmHg) (*p* = 0.0032). Similarly, PP increased significantly from 40 mmHg in T1 to 46 mmHg in T2 and 44 mmHg in T3 (*p* = 0.0020). Total white blood cell count showed a decreasing trend across tertiles: 7.0 × 10^9^/L in T1, 6.7 × 10^9^/L in T2, and 5.5 × 10^9^/L in T3 (*p* < 0.0001). Neutrophils decreased from 4.7 × 10^9^/L in T1 to 2.0 × 10^9^/L in T3, while lymphocytes increased from 2.0 × 10^9^/L in T1 to 2.7 × 10^9^/L in T3 (both *p* < 0.0001). Hemoglobin was significantly higher in T2 (13.5 g/dL) than in T1 (12.9 g/dL) (*p* = 0.0170). HbA1c levels rose from 5.4% in T1 to 5.6% in T3 (*p* = 0.0181). Triglyceride levels rose from 0.94 mmol/L in T1 to 1.09 mmol/L in T3 (*p* = 0.0322), while LDL-C rose from 2.81 mmol/L to 3.32 mmol/L (*p* = 0.0259). Although AIP did not differ significantly across BAN score tertiles, both CRI-I and CRI-II showed significant differences (*p* = 0.0279 and *p* = 0.0018, respectively).

### 3.3. Distribution and Correlation of BAN Score with Cardiometabolic Risk Indicators

**Figure 1:**
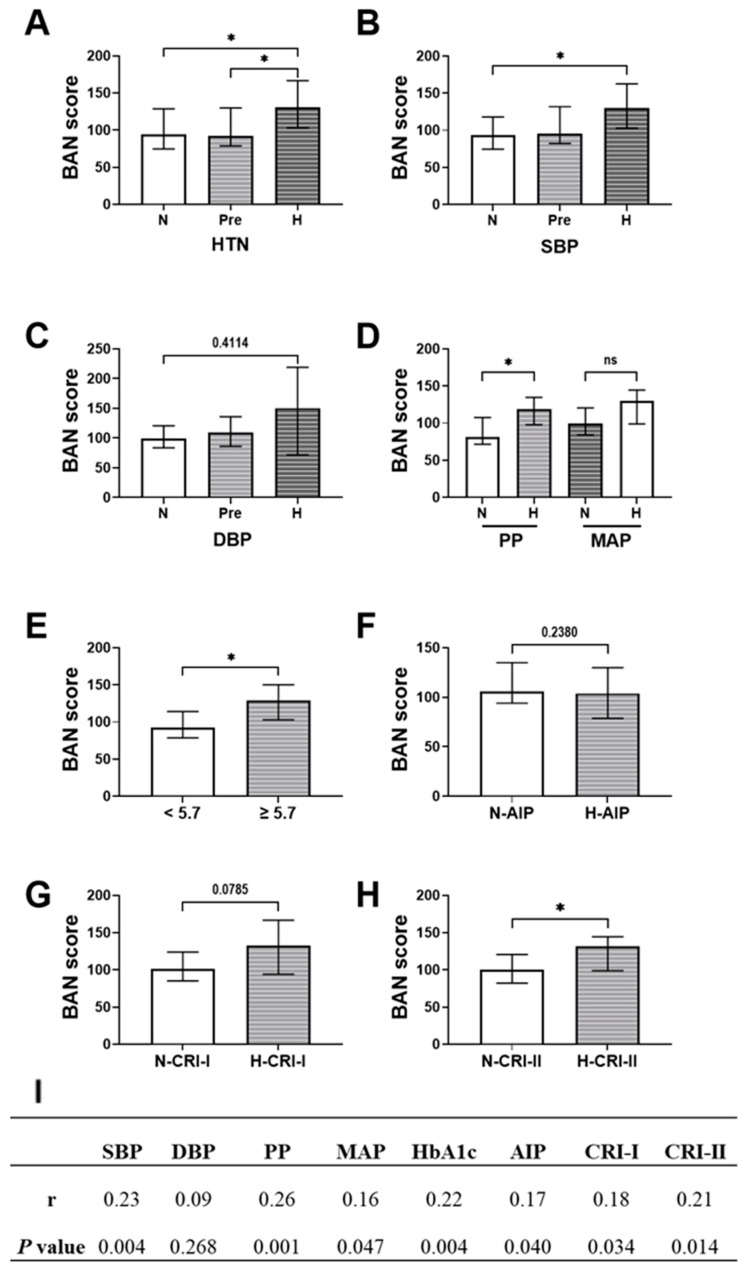
Distribution and correlation of BAN score across different cardiometabolic risk categories. (**A**) BAN score by blood pressure status: normotensive (N), prehypertensive (Pre), hypertensive (H). BAN score stratified by (**B**) systolic blood pressure and (**C**) diastolic blood pressure. (**D**) BAN score levels among individuals with normal and elevated pulse pressure (PP) and mean arterial pressure (MAP). (**E**) BAN score by glycemic status (normal vs. elevated HbA1c). BAN score distribution across normal and high categories of atherogenic lipid indices: (**F**) AIP, (**G**) CRI-I, and (**H**) CRI-II. (**I**) Spearman correlation coefficients between BAN score and cardiometabolic parameters, including SBP, DBP, pulse pressure (PP), MAP, HbA1c, AIP, CRI-I, and CRI-II. Statistical comparisons were conducted using the Kruskal–Wallis test or Kolmogorov–Smirnov test as appropriate. *p* < 0.05 was considered statistically significant. Abbreviations: ns denotes no statistical significance (*p *≥ 0.05), while * denotes *p* < 0.05.

BAN score levels demonstrated a consistent upward trend across categories of increasing cardiometabolic risk. In Figure 1A, median BAN scores were highest in hypertensive individuals (131.3), compared to prehypertensive (92.3) and normotensive participants (94.0). A similar pattern was observed with systolic blood pressure (Figure 1B), where median scores increased significantly from 93.1 in the lowest SBP category to 130.3 in the highest (*p* = 0.0210). Diastolic blood pressure in Figure 1C showed comparable trends, with median BAN scores rising from 99.4 to 150.5 across DBP strata; however, this difference did not reach the level of significance (*p* = 0.3026). Elevated PP was significantly associated with higher BAN scores (median: 119.2 vs. 81.4, *p* = 0.0223), whereas no statistically significant change was detected with MAP, despite a higher median in the elevated group (130.3 vs. 99.4, *p* = 0.2284) (Figure 1D). Individuals with elevated HbA1c exhibited significantly higher BAN scores (Figure 1E, median: 129.4) compared to those with normal HbA1c (Figure 1E, 92.3; *p* = 0.0170), indicating early glycemic stress. Regarding lipid risk indices, individuals with elevated AIP and CRI-I showed higher BAN scores (Figure 1F, median: 136.0 vs. 99.6 for AIP, *p* = 0.0671; and Figure 1G, 132.5 vs. 101.5 for CRI-I, *p* = 0.0785), while not attaining the level of significance. However, BAN scores were significantly elevated among those with high CRI-II values (Figure 1H, 131.3 vs. 100.4; *p* = 0.0238).

Spearman correlation analysis (Figure 1I) revealed that the BAN score was positively correlated with several key cardiometabolic markers. The strongest association was observed with pulse pressure, showing a correlation coefficient of 0.26 (*p* < 0.05), followed by systolic blood pressure with a coefficient of 0.23 (*p* < 0.01) and HbA1c with a coefficient of 0.22 (*p* < 0.01). Significant positive correlations were also noted with the atherogenic lipid indices: AIP (correlation coefficient 0.18), CRI-I (0.18), and CRI-II (0.21), all with *p* < 0.05.

### 3.4. Association of BAN Score with Systolic Load, Pulse Pressure, and Glycemic Status Independent of Several Key Covariates

Linear regression analyses revealed that higher BAN scores were significantly associated with several key cardiometabolic and hemodynamic parameters. As shown in Table 3, higher BAN scores were significantly associated with elevated systolic blood pressure (β = 1.44, 95% CI: 0.58–2.29, *p* = 0.0011) and pulse pressure (β = 0.82, 95% CI: 0.37–1.29, *p* = 0.0005), as well as increased MAP (β = 1.14, 95% CI: 0.21–2.08, *p* = 0.0171) and HbA1c (β = 2.12, 95% CI: 0.76–3.48, *p* = 0.0024) in unadjusted models. BAN score also showed associations with atherogenic indices, including AIP (*p* = 0.042) and Castelli Risk Index-II (*p* = 0.0214). After adjustment for age, sex, smoking, and medication use, BAN remained significantly associated with systolic blood pressure (β = 1.05, 95% CI: 0.06–2.05, *p* = 0.038), pulse pressure (β = 0.66, 95% CI: 0.18–1.15, *p* = 0.002), and HbA1c (β = 1.88, 95% CI: 0.48–3.29, *p* = 0.009), whereas associations with MAP, AIP, and Castelli indices were attenuated and lost significance.

### 3.5. BAN Score Demonstrates Moderate Discriminatory Power for Hypertension and Early Glycemic Risk

In Table 4, receiver operating characteristic (ROC) curve analysis revealed that BAN score achieved an AUC of 0.6650 (*p* = 0.009) for hypertension and 0.6644 (*p* = 0.009) for elevated systolic blood pressure, reflecting fair discriminatory ability in both cases. Similarly, the BAN score demonstrated comparable discriminatory power for elevated HbA1c, yielding an AUC of 0.6644 (*p* = 0.009). However, the BAN score demonstrated a weak, non-significant ability to detect AIP (AUC = 0.5505, *p* = 0.308). Its performance was better and statistically significant for CRI-I (AUC = 0.6207, *p* = 0.011), while CRI-II showed a lower, non-significant AUC of 0.5979 (*p* = 0.115).

### 3.6. Among Individuals with Higher BAN Scores, Hypertension and Elevated Vascular Load Were Observed More Frequently

Risk analysis revealed that both prevalence ratios (PRs) and odds ratios (ORs) showed strong and consistent associations with systolic and pulsatile pressure components, further affirming the clinical relevance of the BAN score in capturing early vascular strain (Table 5). Individuals in higher BAN score strata were significantly more likely to exhibit hypertension. The prevalence ratio for hypertension was 1.55 (*p* = 0.0066), and the corresponding odds ratio was notably elevated at 2.54 (*p* = 0.0147). Systolic blood pressure (SBP) showed a similarly strong relationship with BAN score. The PR for high SBP was 1.58 (*p* = 0.0047), and the OR was 2.69 (*p* = 0.0133), suggesting that increased BAN scores are robustly associated with elevated systolic load. Likewise, pulse pressure (PP), was also significantly associated with higher BAN scores (PR = 1.56, *p* = 0.0275; OR = 2.22, *p* = 0.0189). In contrast, BAN score showed no significant associations with DBP or MAP. Although point estimates suggested elevated risk (e.g., DBP OR = 1.95 and MAP OR = 2.03), wide confidence intervals and *p* values > 0.05 suggest limited predictive utility for these parameters. Elevated HbA1c was associated with a higher prevalence (PR = 1.38; *p* = 0.0486), while the corresponding odds ratio (1.90) showed a borderline significance (*p* = 0.0581). No significant risk elevation was observed for AIP, CRI-I, or CRI-II, as all corresponding PRs and ORs had wide confidence intervals and non-significant *p* values.

## 4. Discussion

This investigation assessed the clinical utility of the BAN score, a composite score integrating body mass index (BMI), serum albumin, and neutrophil-to-lymphocyte ratio (NLR), as a marker of early cardiometabolic dysfunction in a non-diabetic adult population. Individuals in the higher BAN score tertiles exhibited raised systolic blood pressure (SBP), greater pulse pressure (PP), and higher hemoglobin A1c (HbA1c) levels, suggesting early vascular stiffness and glycemic dysregulation. Higher BAN score tertiles were associated with greater atherogenic dyslipidemia, including higher triglycerides and LDL-C, lower HDL-C. Moreover, significant positive correlation was found between the BAN score and SBP, PP, HbA1c, AIP, and CRI-II. In adjusted linear regression models, higher BAN scores remained independently associated with SBP, PP, and HbA1c, reinforcing their link to hemodynamic stress and glycemic elevation. In summary, the BAN score may represent a practical, multifaceted tool for timely identification of vascular dysfunction and dysglycemia, even in non-diabetic individuals. Its strong association with blood pressure and glycemic markers highlights its potential for clinical risk stratification.

As the BAN score combines BMI, serum albumin, and NLR, its association with elevated systolic blood pressure (SBP) and widened pulse pressure (PP) likely reflects interconnected hemodynamic, inflammatory, and metabolic mechanisms. Elevated BMI is known to activate the sympathetic nervous system and enhance renal sodium reabsorption, both of which elevate SBP, particularly when coupled with central arterial stiffening [29,30]. BMI has also been independently linked to arterial stiffness, a key driver of increased SBP and PP [31]. In a large cohort of 62,113 Royal Thai Army personnel, higher BMI was independently associated with progressively higher PP across normal weight, overweight, and obesity categories. Elevated PP was especially prevalent among overweight and obese individuals with otherwise normal blood pressure, underscoring a strong BMI–PP relationship [32]. his interplay partly reflects vascular aging, where elastin degradation and collagen accumulation reduce arterial compliance, producing abnormal increases in SBP and PP [33]. The early return of wave reflections, a hallmark of arterial stiffness, further augments central pressure, particularly SBP and PP [34,35]. These effects may be exacerbated by hypoalbuminemia and systemic inflammation, both integral components of the BAN score. Low serum albumin has been implicated in impaired nitric oxide synthesis, vascular narrowing, sodium accumulation, and widespread endothelial dysfunction, all of which elevate blood pressure [20,36]. Similarly, elevated NLR reflects systemic inflammation, with neutrophil elastase (NE) and neutrophil extracellular traps (NETs) contributing to endothelial injury, vascular remodeling, and arterial stiffening [37,38,39,40]. Together, these mechanisms explain the observed association between BAN score and vascular parameters.

The observed association between the BAN score and HbA1c in our study may reflect underlying inflammatory and metabolic disturbances that link adiposity, nutritional status, and immune activity to early glycemic dysregulation. Elevated BMI, a key component of the BAN score, is a hallmark of obesity, which is closely linked to persistent subclinical inflammation and diminished insulin sensitivity, both central features of metabolic syndrome [41]. Dysfunctional adipose tissue in obesity serves as a source of pro-inflammatory cytokines and adipokines, sustaining chronic inflammation, disrupting insulin signaling pathways, and ultimately impairing glucose homeostasis [42,43]. Adipocytokines such as TNF-α, IL-6, and adiponectin play pivotal roles in mediating insulin resistance and linking obesity to increased cardiovascular risk [44]. Importantly, this inflammatory environment also affects hepatic protein synthesis, leading to reduced serum albumin concentrations, thereby linking hypoalbuminemia, another component of the BAN score to metabolic stress [45]. Hypoalbuminemia not only reflects the presence of inflammation but also contributes to endothelial dysfunction, a known contributor to insulin resistance and vascular complications [46]. Moreover, neutrophils, through the release of NETs, may directly damage pancreatic islet cells and impair insulin secretion, further exacerbating glycemic control [47,48,49,50]. As such, the BAN score may serve as a surrogate for early vascular and metabolic stress that precedes overt diabetes, as reflected by its association with elevated HbA1c levels in our non-diabetic cohort.

The absence of an association between the BAN score and lipid-based cardiometabolic indices such as AIP, CRI-I, and CRI-II may reflect the distinct physiological pathways these measures capture. AIP and CRI ratios are well-recognized markers of chronic dyslipidemia and lipid-driven atherosclerotic risk, representing long-term disturbances in lipid metabolism [51,52,53,54,55]. In contrast, the BAN score integrates serum albumin and NLR, both of which are more indicative of inflammation and nutritional status than lipid imbalance. These components are also more responsive to short-term physiological fluctuations and systemic stress. Recent evidence illustrates this distinction. A NHANES-based and Mendelian randomization study reported that serum albumin was inversely correlated with HDL-C in observational data but causally associated with higher triglycerides, suggesting complex, context-dependent effects on lipid metabolism [56]. Similarly, in a study of dyslipidemic obese children, NLR showed no significant association with lipid abnormalities [57], and in a large rural Chinese cohort, NLR was not significantly linked to metabolic disorders or cardiovascular disease risk [58]. These findings suggest that albumin and NLR may reflect inflammatory and metabolic stress pathways rather than lipid-specific mechanisms. Overall, while BMI strongly associates with lipid disturbances, albumin and NLR do not, limiting the BAN score’s ability to capture lipid-driven risk. This highlights the multidimensional nature of cardiometabolic risk and the need for complementary indices.

These findings carry important clinical implications, with the BAN score offering a practical, low-cost, and routinely accessible tool to support early identification of individuals at risk for cardiometabolic dysfunction, particularly in primary care and resource-constrained settings. Although the BAN score has been primarily studied in cancer-related prognosis [17,18], the present study underscores its potential relevance in metabolic and cardiovascular risk assessment. Rather than replacing established risk markers, BAN should be considered a complementary index that broadens the information available for clinical evaluation. At present, no standardized thresholds exist for BAN in cardiometabolic contexts. In this study, tertile-based stratification was applied to demonstrate graded associations, but such statistical cut-points are not equivalent to clinically actionable values. Moving toward clinical application will require determining thresholds that optimize sensitivity and specificity for outcomes such as hypertension, arterial stiffness, and glycemic dysregulation. This should be pursued in large, prospective, multi-ethnic cohorts, ideally using ROC analyses and survival modeling to establish prognostic value. Threshold selection may also need to account for sex- or age-specific differences, as well as modification by comorbidities or pharmacologic treatments. Importantly, because this study excluded patients with diabetes and overt CVD, the distribution and predictive ability of BAN in these populations remain unknown. It is likely that BAN values would be higher and predictive patterns influenced by disease burden and therapies, highlighting the need for dedicated investigations in these groups. Ultimately, establishing robust cut-offs could enable BAN to be integrated into existing cardiovascular risk stratification algorithms, supporting earlier interventions and targeted preventive strategies.

This research is strengthened by the first-time application of the BAN score in a non-diabetic population and the broad analysis of its associations with multiple cardiometabolic measures. Importantly, the reliance on routinely collected parameters enhances feasibility in real-world practice. Nonetheless, several limitations must be acknowledged. First, the cross-sectional design restricts interpretation to associations rather than causality, and reverse causation cannot be excluded. Second, the single-center origin of the cohort may limit generalizability across diverse populations and clinical settings. Third, lifestyle and behavioral factors such as diet and physical activity, were not available, which may contribute to residual confounding. Finally, we lacked refined adiposity measures such as waist circumference, waist-to-hip ratio, or direct body fat percentage, which may have provided additional mechanistic insight. These limitations should be considered when interpreting the findings, and future longitudinal, multi-center studies with richer phenotyping are needed to validate and extend our results.

## 5. Conclusions

In conclusion, the BAN score, originally designed as a nutrition–inflammation index in oncology, demonstrated significant associations with systolic hypertension, widened pulse pressure, and impaired glucose regulation in a non-diabetic adult cohort. These findings suggest that BAN may serve as a simple and integrative biomarker of early cardiometabolic stress, with the advantage of being derived from routinely available clinical parameters. Importantly, this work extends the application of BAN beyond its original oncological context and highlights its potential relevance in primary prevention of cardiovascular disease. Nevertheless, the cross-sectional and single-center design, as well as the lack of detailed lifestyle and adiposity measures, limit both causal inference and generalizability. Future longitudinal and multi-center studies are warranted to confirm its prognostic value, define clinically meaningful cut-off thresholds, and test whether BAN-guided interventions could improve patient outcomes. If validated, BAN could provide a cost-effective, accessible tool for early risk stratification and targeted prevention strategies in broader clinical practice.

## Figures and Tables

**Table 1:** Participant characteristics and comorbidities.

Variable	Median ± IQR or n (%)
Continuous variables	
Age	37 (28–45)
BMI	33 (27–36)
Albumin	4.4 (4.2–4.6)
NLR	1.3 (0.87–1.92)
WBC	6.4 (5.3–7.8)
Categorical variables	
Sex (Female)	118 (72.8%)
Hypothyroidism	21 (13.0%)
Polycystic Ovary Syndrome (PCOS)	9 (5.6%)
Anti-HTN medications	11 (6.8%)
Smokers	16 (9.9)
Iron Supplementation	35 (21.6)
GLP-1 Receptor Agonists	28 (17.3)

**Table 2:** Clinical, hematological, and biochemical variables according to BAN score tertiles.

Variable	T1	T2	T3	*p* Value
Age	35 (26–43)	38 (28–44)	38.0 (29–50)	0.1844
SBP (mmHg)	117 (107–123)	124 (115–131)	120 (111–130)	0.0032
DBP (mmHg)	75 (70–80)	77.0 (71–84)	75 (70–81)	0.2576
PP (mmHg)	40 (34–46)	46 (40–51)	44 (39–51)	0.0020
MAP(mmHg)	89 (81–94)	93 (87–99)	90 (83–96)	0.0596
WBC × 10^9^/L	7.0 (5.9–9.0)	6.7 (6.0–7.8)	5.5 (4.6–6.4)	<0.0001
RBC × 10^12^/L	4.7 (4.4–5.1)	4.9 (4.7–5.4)	4.8 (4.5–5.3)	0.0668
Hb (g/dL)	12.9 (11.3–14.0)	13.5 (12.6–15.4)	13.3 (12.2–14.3)	0.0170
Hct (L/L)	0.39 (0.37–0.42)	0.42 (0.39–0.46)	0.42 (0.38–0.44)	0.0119
MCV (fL)	86.0 (80.9–88.5)	85.0 (81.0–89.0)	84.5 (79.0–88.0)	0.8424
MCH (pg)	27.9 (25.8–28.9)	28.0 (26.3–29.0)	27.65 (25.1–29.4)	0.8126
Platelet (×10^9^/L)	321 (273–348)	299 (253–355)	286 (253–322)	0.1853
Neutrophil(×10^9^/L)	4.7 (3.5–5.6)	3.2 (2.7–4.0)	2.0 (1.5–2.7)	<0.0001
Lymphocytes (×10^9^/L)	2.0 (1.7–2.5)	2.5 (2.2–2.9)	2.7 (2.3–3.1)	<0.0001
Monocytes (×10^9^/L)	0.5 (0.4–0.5)	0.5 (0.4–0.6)	0.4 (0.3–0.5)	0.0310
Eosinophile (×10^9^/L)	0.13 (0.07–0.20)	0.19 (0.12–0.28)	0.14 (0.1–0.19)	0.0058
Basophile (×10^9^/L)	0.04 (0.02–0.06)	0.04 (0.03–0.06)	0.04 (0.03–0.05)	0.6947
HbA1c (%)	5.4 (5.2–5.6)	5.6 (5.2–5.8)	5.6 (5.3–5.9)	0.0181
TC (mmol/L)	4.71 (3.97–5.43)	5.04 (4.49–5.59)	5.07 (4.36–5.86)	0.2639
TG (mmol/L)	0.94 (0.63–1.20)	1.18 (0.85–1.61)	1.09 (0.80–1.79)	0.0322
HDL (mmol/L)	1.44 (1.15–1.68)	1.23 (1.05–1.54)	1.22 (1.04–1.5)	0.0903
LDL (mmol/L)	2.81 (2.48–3.36)	3.15 (2.72–3.95)	3.32 (2.73–3.95)	0.0259
AIP	0.21 (0.02–0.32)	0.27 (0.15–0.51)	0.24 (0.13–0.56)	0.1001
CRI-I	1.57 (1.02–2.02)	2.01 (1.45–3.42)	1.67 (1.35–3.65)	0.0279
CRI-II	2.03 (1.72–2.50)	2.73 (2.09–3.38)	2.51 (1.92–3.36)	0.0018

Abbreviations: SBP = Systolic Blood Pressure; DBP = Diastolic Blood Pressure; PP = Pulse Pressure; MAP = Mean Arterial Pressure; WBC = White Blood Cell Count; RBC = Red Blood Cell Count; Hb = Hemoglobin; Hct = Hematocrit; MCV = Mean Corpuscular Volume; MCH = Mean Corpuscular Hemoglobin; HbA1c = Glycated Hemoglobin; TC = Total Cholesterol; TG = Triglycerides; HDL-C = High-Density Lipoprotein Cholesterol; LDL-C = Low-Density Lipoprotein Cholesterol; AIP = Atherogenic Index of Plasma; CRI-I/II = Castelli Risk Index I/II; IQR = Interquartile Range.

**Table 3:** Linear regression analysis for BAN score with other parameters.

	Model 1		Model 2	
Variable	β (95% CI)	*p* Value	β (95% CI)	*p* Value
Systolic Blood Pressure	1.44 (0.58 to 2.29)	0.0011	1.05 (0.06 to 2.05)	0.038
Diastolic Blood Pressure	0.62 (−0.25 to 1.50)	0.1631	0.08 (−0.88 to 1.04)	0.875
Pulse Pressure	0.82 (0.37 to 1.29)	0.0005	0.66 (0.18 to 1.15)	0.002
Mean Arterial Pressure	1.14 (0.21 to 2.08)	0.0171	0.59 (−0.49 to 1.67)	0.279
Glycated Hemoglobin A1c	2.12 (0.76 to 3.48)	0.0024	1.88 (0.48 to 3.29)	0.009
Atherogenic Index of Plasma	0.35 (0.01 to 0.68)	0.042	0.10 (−0.07 to 0.262)	0.261
Castelli Risk Index-I	0.13 (−0.01 to 0.28)	0.0762	0.30 (−0.06 to 0.67)	0.103
Castelli Risk Index-II	0.32 (0.05 to 0.59)	0.0214	0.29 (−0.01 to 0.59)	0.052

Note: Model 1 represented the unadjusted analysis, and Model 2 accounted for age, sex, smoking status and medication use as covariates.

**Table 4:** Discriminatory ability of BAN score to identify cardiometabolic risk markers.

Variable	AUC	Std. Error	95% Confidence Interval	*p*-Value
Hypertension	0.6650	0.0586	0.5502–0.7798	0.009
Systolic Blood Pressure	0.6644	0.0524	0.5618–0.7670	0.009
Diastolic Blood Pressure	0.6574	0.0782	0.5042–0.8105	0.138
Pulse Pressure	0.6207	0.6207	0.5286–0.7127	0.011
Mean Arterial Pressure	0.5979	0.0515	0.4970–0.6987	0.115
Glycated Hemoglobin A1c	0.6644	0.0525	0.5618–0.7670	0.009
Atherogenic Index of Plasma	0.5505	0.0493	0.4539–0.6472	0.308
Castelli Risk Index-I	0.6207	0.0497	0.5286–0.7127	0.011
Castelli Risk Index-II	0.5979	0.0515	0.4970–0.6867	0.115

**Table 5:** Risk estimates for cardiometabolic outcomes according to BAN score levels.

Variable	PR	95% CI	*p* Value	OR	95% CI	*p* Value
Hypertension	1.55	1.13–2.13	0.0066	2.54	1.20–5.36	0.0147
Systolic Blood Pressure	1.58	1.15–2.17	0.0047	2.69	1.23–5.89	0.0133
Diastolic Blood Pressure	1.36	0.77–2.38	0.2883	1.95	0.45–8.46	0.3712
Pulse Pressure	1.56	1.05–2.33	0.0275	2.22	1.14–4.33	0.0189
Mean Arterial Pressure	1.40	0.98–2.00	0.0675	2.03	0.86–4.81	0.1059
Glycated Hemoglobin A1c	1.38	1.00–1.91	0.0486	1.90	0.98–3.69	0.0581
Atherogenic Index of Plasma	1.02	0.72–1.43	0.9299	1.03	0.53–2.01	0.9299
Castelli Risk Index-I	1.27	0.86–1.87	0.2383	1.64	0.67–3.99	0.2776
Castelli Risk Index-II	1.27	0.89–1.82	0.1821	1.63	0.76–3.49	0.2055

## Data Availability

The data that support the findings of this study were obtained from the institutional database of Prince Sultan Military Medical City (PSMMC), Riyadh, Saudi Arabia. Due to ethical and legal restrictions related to patient confidentiality, these data are not publicly available. De-identified datasets may be made available from the corresponding author upon reasonable request and with permission from the PSMMC Institutional Review Board.
